# Genetic alteration of Chinese patients with rectal mucosal melanoma

**DOI:** 10.1186/s12885-021-08383-6

**Published:** 2021-05-27

**Authors:** Huan Li, Lujing Yang, Yumei Lai, Xintong Wang, Xinyin Han, Siyao Liu, Dongliang Wang, Xiaojuan Li, Nana Hu, Yan Kong, Lu Si, Zhongwu Li

**Affiliations:** 1 Key Laboratory of Carcinogenesis and Translational Research (Ministry of Education), Department of Pathology, Peking University Cancer Hospital & Institute, Fucheng Road No.52, Haidian District, Peking, 100142 Beijing, People’s Republic of China; 2 ChosenMed Technology (Beijing) Co., Ltd., Beijing, 100176 People’s Republic of China; 3 Computer Network Information Center, Chinese Academy of Sciences, Beijing, 100190 People’s Republic of China; 4 University of the Chinese Academy of Sciences, Beijing, 100190 People’s Republic of China; 5 Key Laboratory of Carcinogenesis and Translational Research (Ministry of Education), Department of Renal Cancer and Melanoma, Peking University Cancer Hospital & Institute, Fucheng Road No.52, Haidian District, 100142 Beijing, People’s Republic of China

**Keywords:** RMM, Prognosis, *BRAF* mutations, *NRG1* deletions

## Abstract

**Background:**

Rectal mucosal melanoma (RMM) is a rare and highly aggressive disease with a poor prognosis. Due to the rarity of RMM, there are few studies focusing on its genetic mechanism. This retrospective study aimed to analyze the genetic spectrum and prognosis of RMM in China and lay a foundation for targeted therapy.

**Methods:**

36 patients with primary RMM from Peking University Cancer Hospital were enrolled in this study. The Next-generation sequencing (NGS) data of the tumor samples were fitted into the TruSight™ Oncology 500 (TSO500) Docker pipeline to detect genomic variants. Then, the univariate and multivariate Cox hazard analysis were performed to evaluate the correlations of the variants with the overall survival (OS), along with Kaplan-Meier and log-rank test to determine their significance.

**Results:**

*BRAF* mutations, *NRG1* deletions and mitotic index were significant prognostic factors in the univariate analysis. In multivariable analysis of the OS-related prognostic factors in primary RMM patients, it revealed 2 significant alterations: *BRAF* mutations [HR 7.732 (95%CI: 1.735–34.456), *P* = 0.007] and *NRG1* deletions [HR 14.976 (95%CI: 2.305–97.300), *P* = 0.005].

**Conclusions:**

This is the first study to show genetic alterations exclusively to Chinese patients with RMM. We confirmed genetic alterations of RMM differ from cutaneous melanoma (CM). Our study indicates that *BRAF* and *NRG1* were correlated with a poor prognostic of RMM and may be potential therapeutic targets for RMM treatment.

**Supplementary Information:**

The online version contains supplementary material available at 10.1186/s12885-021-08383-6.

## Introduction

Mucosal melanoma (MM) is a malignant tumor caused by the aberrant growth of melanocytes in the mucosal lining. As a subtype of melanoma, MM may occur in the mucosal layer of different anatomical parts, such as the respiratory tract, gastrointestinal tract and urogenital tract. Compared with cutaneous melanoma (CM), MM is particularly rare. In the United States, MM accounts for only 1.3% of melanoma, while melanoma originated from skin accounts for 91.2% [1]. However, in a study enrolling 522 Chinese melanoma patients, the incidence of MM was 22.6% [2]. Anorectal mucosal melanoma (ARMM) is one of the most common types of MM, often accompanied by a less favorable prognosis [3]. Although melanoma occurring in rectum is more common than that in anus, most studies always combine these two entities [4, 5]. In addition, Tchelebi et al. showed that the incidence of rectal mucosal melanoma (RMM) continued to rise, with a higher rate of growth than anal melanoma over the past decades [6]. However, whether the lesion is located in anus or rectum, the long-term prognosis is poor [7]. Due to the low incidence of the disease and less reported, the etiology, pathogenesis and genetic cognition of RMM are still unclear, which brings difficulties to the correct diagnosis, treatment and prognosis prediction.

So far, surgery has been recommended as the optimal treatment for this rare tumor, allowing for complete local excision [8, 9]. The additional radiotherapy does not improve prognosis of patients with RMM, even those with more locally advanced disease [6]. Chemotherapy and biochemotherapy showed limited efficacy on MM patients, while there was no significant survival improvement in CM patients [10]. Despite surgical resection and emergence of various forms of adjuvant therapy, the overall prognosis of RMM remains dismal [11]. For CM, targeted therapy and immunotherapy have been shown to improve prognosis in advanced patients [12]. However, at present, the applicability of targeted treatment based on *BRAF* inhibitors is limited because the incidence of *BRAF* mutations in MM is much lower than that in CM [13]. Therefore, the discovery of potential therapeutic targets of RMM is particularly important.

It is well documented that mutational profiles of malignant melanomas in China are significantly different from that in Western countries [14, 15]. A genome-wide landscape study also confirmed that MM showed different mutation characteristics in different anatomical parts [16]. Therefore, we analyzed genetic alterations of 36 Chinese primary RMM verifying the difference of alterations between MM and CM. In addition, we revealed that *BRAF* and *NRG1* could be potential therapeutic targets for RMM. Our study depicted the mutation landscape of Chinese RMM and provides more insights into its targeted treatment.

## Method

### Patients and pathology

We enrolled 36 RMM patients treated in Peking University Cancer Hospital from May 2010 to March 2019, along with their clinical and sequencing data. This retrospective study was approved by the ethics committee of Peking University Cancer Hospital. All patients have signed the informed consent. Clinical data included age at diagnosis, sex, treatment and follow-up results. All cases have formalin-fixed and paraffin-embedded (FFPE) samples available for analyses and they were confirmed diagnosed by 2 experienced pathologists in the pathology department. The following primary tumor clinicopathological characteristics were assessed: depth of tumor invasion (lamina propria, submucosa, muscularis propria or serosa and beyond); pigment content; thickness; vascular invasion; perineural invasion; ulcer of primary focus; mitotic index and lymph node metastasis (Table 1).

**Table 1:** Clinicopathological parameters of 36 primary RMM patients

Variables	n	%
**Age, years**		
Median	62 (43–98)	
**Sex**		
Male	14	38.9
Female	22	61.1
**Depth of tumor invasion**		
Lamina propria	0	0
Submucosa	10	27.8
Muscularis propria	20	55.6
Perirectal soft tissue	6	16.6
Serosa and beyond	0	0
**Pigment content**		
High	13	36.1
Low	14	38.9
No	6	16.7
Unknown	3	8.3
**Thickness, mm**		
Median	11	
**Vascular invasion**		
Absent	19	52.8
Present	17	47.2
**Perineural invasion**		
Absent	25	69.4
Present	11	30.6
**Ulceration**		
Absent	6	16.7
Present	30	83.3
**Mitotic count,**		
Median	16	
**Lymph node metastasis**		
Absent	12	33.3
Present	22	61.1
Unknown	2	5.6

### Immunohistochemistry (IHC) staining

Common pathological markers of melanoma including CK, SOX-10, Melan-A, HMB-45 and S-100 were identified by IHC staining. The IHC results were independently evaluated by two different pathologists, who did not know the clinical information related to the subjects. If the two pathologists got different results, a third pathologist would review the slide. For SOX-10, IHC staining of any tumor cell nucleus was positive; for HMB-45, Melan-A and S-100, IHC staining was positive when any tumor cells showed cytoplasmic staining, and for CK, IHC staining was negative when all tumor cells showed loss of membrane staining. The tumor was considered to be MM if at least one staining of Melan-A, HMB-45, S-100, and SOX-10 was positive and CK was negative.

### DNA and RNA extractions

Genomic DNA and RNA samples were extracted from the same FFPE tissue sections using kit AllPrep DNA/RNA FFPE KIT (Cat No./ID:80234) according to the manufacturer’s protocols.

In this study, the preparation of both RNA and DNA sample library were performed according to the TSO500 Library Preparation Kit (Illumina, San Diego, CA, USA). Then, a two-step capture and enrichment of specific capture probes for DNA samples was performed, the DNA library and corresponding cDNA library of 8 samples was standardized by using the library homogenization method based on magnetic bead purification and sequenced using the Illumina NextSeq 550Dx platform. The Sequencing results were analyzed using TSO500 Docker pipeline.

### Sequence data processing

The sequence alignment to the human genome (hg19) was completed using the BWA-MEM (version 0.7.11) [17] alignment algorithm. SAMtools (version 1.3) [18] was used to do bam-sam conversions. We used Genome Analysis Toolkit (GATK, version 3.6) [19] module IndelRealigner was used to perform local realignment of indels. Germline variants were filtered out using in-house built database, and all parameter were set according to [20]. Copy number variants (CNVs) including amplification and deletion were identified by CRAFT copy-number callers from the TSO500 pipeline. Manta (version 1.6.0) [21] was employed to detect large-scale structure variations (SVs) in RNA library, and only fusions having at least 3 unique supporting reads, one of which being a split read crossing the fusion breakpoint, are considered as candidate fusions.

### Measurement of immunotherapy biomarkers

Tumor mutation burden (TMB) was measured as the number of eligible somatic mutations (coding or high-confidence regions, over 50x coverage, over 5% VAF single-nucleotide variants (SNVs) and indels) by NGS per Megabase (Mb), after filtering germline variants (in-house database) and high COSMIC database counts mutations. Only the SNVs and indels in the coding regions were considered for TMB measurement. Assessment of raw sequencing data and TMB were conducted with the Illumina TSO500 supporting software. TMB-High and TMB-Low were divided by the median TMB score of the 36 RMM patients.

Samples’ microsatellite status was determined using TSO500 Docker pipeline in the form of microsatellite instability (MSI) score. Samples with MSI score ≥ 20 were considered as MSI, otherwise microsatellite stability (MSS).

### Statistical analysis

The primary endpoint of our study was overall survival (OS). OS is defined as the time interval between the date of tumor resection and date of death from any cause or the date of the last follow-up. SPSS software (SPSS, version 20.0) was used to perform statistical analysis. The OS rate was described using Kaplan-Meier survival curves and log-rank test. The Cox proportional hazards model was used to perform univariate and multivariate analysis. And the hazard ratio and 95% confidence interval (95%CI) were recorded for parameters. The statistical significance level was set at 0.05.

## Result

### Clinicopathological characteristics

A total of 36 patients with RMM were included in this study. Our clinicopathological analyze was predominantly based on the primary cases. The male/female ratio was 1:1.57, and the median age was 62 years (range, 43–98 years). Other distribution of relevant parameters of 36 primary RMM patients was summarized in Table 1.

A majority of RMM patients had lymph node metastasis (61.1%), while 33.3% RMM did not occur and 5.6% remained undetermined. As RMM is not included in the American Joint Committee on Cancer (AJCC) melanoma staging system, we used the depth of tumor invasion which is similar to the T staging used in colorectal cancer. The level of invasion for the non-perianal tumors was either submucosal (10/36, 27.8%), to muscularis propria (20/36, 55.6%) and beyond the muscularis propria into perirectal soft tissue (6/36, 16.6%). It is worth noting that most of patients presented with ulcers (83.3%). Vascular and perineural invasion were identified in 17 (47.2%) and 11 (30.6%) tumors, respectively. The median thickness of the tumor is 11 mm.

### Mutation profile of RMM

We performed NGS on tumor samples from 36 RMM patients and assessed the SNVs and the CNVs. There were 183 gene mutations found in 36 RMM samples, with an average TMB of 9.15 mut/Mb and all patients were assessed as MSS.

**Fig. 1:**
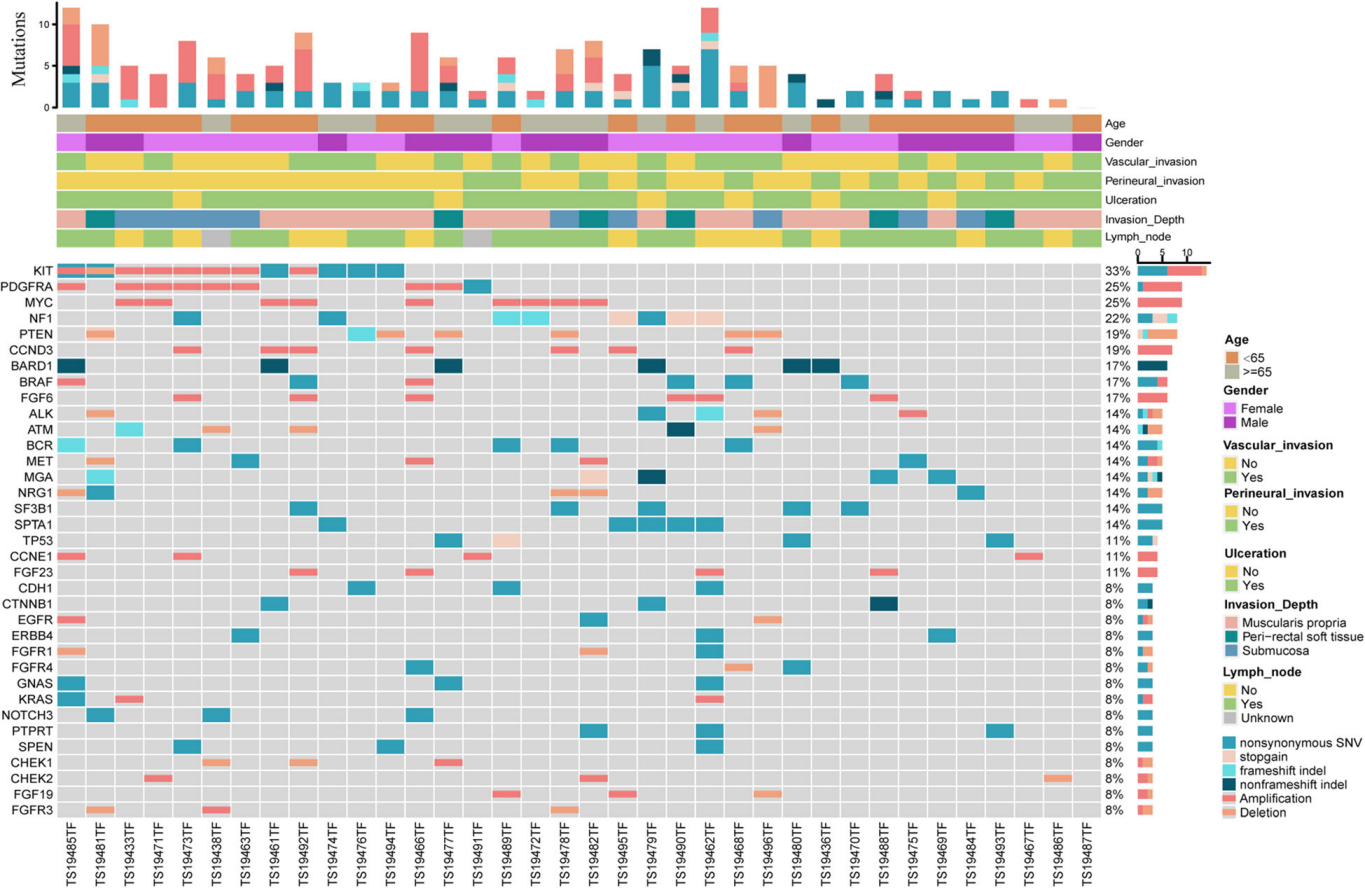
Mutation landscape of 36 RMM (top 35). All somtatic mutations or CNVs that occurred in 3 or more samples were depicted in the figure, along with TMB and clinical information

All the mutant genes were listed in Table S1, while Fig. 1 depicted the mutation profile of top 35 genes. Our results showed that *KIT* was the most frequently mutated gene (33%) in RMM, and there were 5 patients with *KIT* mutation at site p.L576P, one of which had concomitant mutation at site p.W557R. In addition, *BRAF* mutations were diverse, with only one p.V600E mutation and the remaining three mutations were p.D594G, p.G469A, p.R146Q. *SF3B1* were identified in 5 patients (13.9%), 3 of whom showed hotspot mutation in R625H. Other melanoma-related genes were also discovered in RMM mutations, including *TP53* (13.8%) and *NRAS* (2.8%). Besides, genes with moderate mutation frequency were observed, such as *SPTA1*, *BCR*, *MGA*, *GNAS* and *BARD1* (Figure S1).

The top 8 frequent CNVs were depicted in Fig. 1, and more details were in Table S2. In our study, *MYC* (9/36, 25.0%), *CCND3* (7/36, 19.4%) and *PDGFRA* (8/36, 22.2%) showed the most frequent amplification/deletion rates. *KIT* amplifications were detected in 19.4% of cases, mostly on chromosome 4. Interestingly, *KIT* p.L576P mutation existed simultaneously with amplification in one case. *BRAF* amplification was detected in two cases. In addition, we also observed copy number deletions, including *PTEN* (16.7%) and *NRG1* (8.3%). Our data suggested that there were also gene fusions in RMM patients, where one *NTRK* gene fusion and one *BRAF* gene fusion were identified.

### Identification of OS-related markers

We analyzed the association between patients’ OS with clinicopathological characteristics. Of all 36 patients, 12 were alive at the end of follow-up. Prognosis was poor for all patients, with median OS of only 15.7 months (25–75% quartiles: 11.3 months – 40.8 months). Mitotic index (the median of mitotic index served as the threshold value for grouping) and lymph node metastasis were significant in univariate analysis related to OS (*P* = 0.044 and *P* = 0.045, respectively), while other clinicopathological characteristic showed no significant differences (Table S3).

**Fig. 2:**
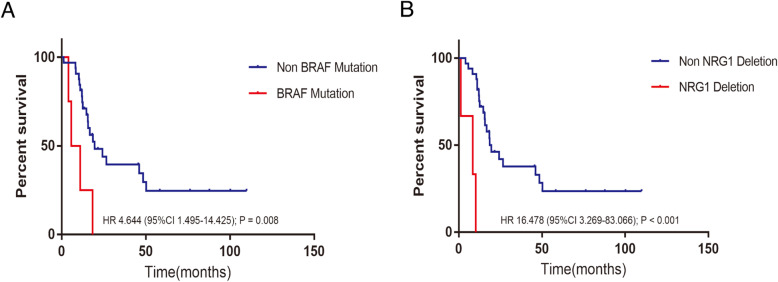
Kaplan-Meier survival curves of OS. **A** BRAF mutations was significantly associated with poor OS (*P* = 0.008). **B** NRG1 CNVs was significantly associated with poor OS (*P* = 0.001)

In order to evaluate the relationship between gene alterations and the survival of MM patients, a gene with mutation frequency or CNVs frequency greater than 8.0% was selected for survival analysis. As a result, we found that patients with *BRAF* mutation had significantly shorter OS than non-mutated ones (*P* = 0.008, Table 2, Fig. 2A). Besides, *NRG1* copy number deletion was present in a total of 8.3% of the cohort and its correlation with OS remained significant (*P* = 0.001, Table 2, Fig. 2B), while other alterations (*KIT*, *SF3B1*, *NF1* and *TP53*) did not show the same performance (Table 2).

**Table 2:** Univariate analysis to identify OS-related genes

Type	Genes	Occurrence (%)	HR(95%CI)	*P*-value
**Mutation**	*NF1*	22.2%	0.418 (0.141–1.238)	0.115
**Mutation**	*KIT*	16.7%	1.387 (0.548–3.513)	0.490
**Mutation**	*BARD1*	16.7%	1.217 (0.415–3.570)	0.721
**Mutation**	*SPTA1*	13.9%	0.565 (0.166–1.918)	0.360
**Mutation**	*MGA*	13.9%	1.619 (0.551–4.756)	0.381
**Mutation**	*SF3B1*	13.9%	1.510 (0.514–4.433)	0.453
**Mutation**	*BCR*	13.9%	0.565 (0.166–1.918)	0.360
**Mutation**	*TP53*	11.1%	1.971 (0.653–5.952)	0.229
**Mutation**	*BRAF*	11.1%	4.644 (1.495–14.425)	**0.008**
**Mutation**	*GNAS*	8.3%	1.570 (0.461–5.349)	0.471
**Mutation**	*CTNNB1*	8.3%	1.057 (0.246–4.535)	0.941
**Mutation**	*SPEN*	8.3%	0.518 (0.120–2.226)	0.376
**Mutation**	*CDH1*	8.3%	0.923 (0.271–3.138)	0.898
**Mutation**	*PTPRT*	8.3%	2.108 (0.618–7.195)	0.234
**Mutation**	*NOTCH3*	8.3%	0.266 (0.036–1.983)	0.196
**Mutation**	*ERBB4*	8.3%	0.604 (0.141–2.585)	0.496
**Amplification**	*MYC*	25.0%	1.168 (0.432–3.227)	0.764
**Amplification**	*PDGFRA*	22.2%	0.490 (0.146–1.648)	0.249
**Amplification**	*CCND3*	19.4%	0.693 (0.236–2.036)	0.505
**Amplification**	*KIT*	19.4%	0.688 (0.199–2.248)	0.515
**Amplification**	*FGF6*	16.7%	0.856 (0.290–2.523)	0.778
**Amplification**	*FGF23*	11.1%	1.005 (0.296–3.413)	0.994
**Amplification**	*CCNE1*	11.1%	1.046 (0.310–3.526)	0.942
**Deletion**	*PTEN*	16.7%	1.655 (0.655–4.183)	0.287
**Deletion**	*NRG1*	8.3%	16.478 (3.269–83.066)	**0.001**

*RMM* rectal mucosal melanoma, *HR* hazard ratio, *CI* confidence interval

Statistically significant *P*-values are bolded

The multivariate model was employed to evaluate the comprehensive prognostic value of features obtained from univariate analysis, as shown in Table 3. It revealed 3 significant prognosis-related variables: lymph node metastasis, *BRAF* mutations and NRG1 deletions, where the significance level for lymph node metastasis was much close to 0.05, and limited to the number of samples, we did not perform an in-depth analysis on it. The overview of our analysis was demonstrated in Figure S2.

**Table 3:** Multivariable analysis to identify OS-related factors

Variable	HR(95%CI)	***P***-value
**Depth of tumor invasion**		0.992
Submucosa	1	
Muscularis propria	0.942 (0.237–3.745)	
Perirectal	1.023 (0.203–5.158)	
**Mitotic index**		0.283
≤ 16	1	
> 16	1.819 (0.610–5.427)	
**Lymph node metastasis**		**0.045**
Absent	1	
Present	3.319 (1.026–10.737	
Unknown	–	
**Ulceration**		0.084
Absent	1	
Present	3.954 (0.830–18.842)	
***NRG1*** **Deletion**		**0.005**
No	1	
Yes	14.976 (2.305–97.300)	
***BRAF*** **Mutation**		**0.007**
No	1	
Yes	7.732 (1.735–34.456)	

Factors considered in multivariable analysis were those with *P-*value < 0.1 in the result of univariate analysis

*HR* hazard ratio, *CI* confidence interval

Statistically significant *P*-values are bolded

## Discussion

In a retrospective cohort involving 446 Chinese patients with melanoma, melanoma was shown to be more aggressive and had a shorter OS than cutaneous lesions [22]. Although similar research has been reported in previous studies, no comprehensive analysis of specific mucosal site has been carried out [15, 23, 24]. In this study, NGS technology was applied to depict the mutation spectrum of 36 RMM patients to provide a reference for the clinical prognosis and further targeted intervention therapy.

A review of the histopathology revealed multiple adverse prognostic pathological factors including deep tumor thickness, ulceration, high tumor mitotic rate and lymphovascular invasion [25]. In our study, univariate analysis confirmed that mitotic index and lymph node metastasis may be risk factors of prognostic significance. Apart from this, Nagarajan et al. found the presence of lymphovascular invasion was correlated with shorter disease-specific survival in ARMM [26], which was also consistent with our research.

Similar to the previous findings [15, 27–29], we identified recognized mutations associated with MAPK signaling pathway, including mutations in *NF1*, *KIT*, *NRAS* and *BRAF*. This provides a reliable theory for targeted therapy of RMM. In the meantime, the mutation profile also suggested that RMM’s mutation characteristic may be different from that of CM [23]. It is well known that *BRAF* mutations occur in more than 50% of CM, and targeted therapy for melanoma has been applied clinically which achieved good efficacy. However, *BRAF* mutations in MM are less frequent than that in CM. A review about *BRAF* mutations collected in 1339 cases of mucosal melanoma revealed that *BRAF* mutations were present in 8.0% (107/1339) MM [13]. In two studies focusing on MM molecular spectrum, the frequency of *BRAF* mutations were reported as 3.1% (2/65) [30] and 7.5% (3/40) [31], respectively. These results are similar to ours (11.1%, 4/36). Moreover, there are significant differences between CM and MM in the location of *BRAF* mutations. It has been documented that small molecule inhibitors (vemurafenib and dabrafenib) of *BRAF* V600–mutant induce tumor regression, and combination of *BRAF* and *MEK* inhibitors can improve survival rate of melanoma patients [32–34].

Although *BRAF* mutations are rare in MM, they are characterized by a higher prevalence of non-V600 mutations than in CM [13], which was also confirmed in our study. It is notable that although the cohort was small, the SNV of *BRAF* was associated with the poor prognosis. Currently, only few studies have investigated the molecular mechanisms of non-*BRAF* V600 mutations, which are able to promote *MEK* phosphorylation in a *CRAF*-dependent manner by directly binding to and activating *CRAF* to drive the MAPK pathway [35]. In consistent with the report of genetic alterations in ARMM [26, 28, 36], other genes such as *SF3B1* and *TP53*, also mutated in our cohort. Apart from these common mutations, many genes had intermediate mutation frequencies in RMM, including *BARD1*, *SPTA1*, *MGA*, and *BCR*. These genes are associated with the occurrence of other malignancies, but have not been reported in MM, indicating that the potential therapeutic targets for RMM needs to be further studied.

Our results revealed that most RMM patients have CNVs mutations, and *MYC* was most frequently amplified, which was consistent with previous melanoma studies [31, 37–40]. High *MYC* expression is often associated with tumor metastasis and poor prognosis in melanoma [41]. However, we did not observe a significant difference of OS between *MYC* amplification and *MYC* non-amplification cohorts. In addition, *PDGFRA*, *CCND3*, *KIT* amplification and *PTEN* deletion have been reported in previous studies of MM [30, 38], which was consistent with our findings. Deletion of the *NRG1* gene was detected in 8.3% (3/36) of RMM patients and was associated with poor prognosis in our cohort. Neuregulins (*NRGs*) are a large subclass of polypeptide growth factors of the epidermal growth factor (*EGF*) family [42]. On one hand, it can specifically bind to the extracellular domain the receptor tyrosine kinase *ERBB3* and *ERBB4* to alter receptor conformation and promote dimerization with *ERBB2* [42]. Receptor hetero dimerization promotes autophosphorylation of the cytoplasmic tyrosine residues, resulting in the activation of downstream PI3 kinase and MAP kinase signaling pathways [43]. *NRG1/ERBB3* signaling was able to negatively regulate melanocyte (MC) differentiation and pigmentation while promoting proliferation [43]. On the other hand, the *NRG1* gene is frequently silenced by methylation in breast cancers and *NRG1* may be the principal tumor suppressor gene that leads to loss of the short arm of chromosome 8 in many breast and other epithelial cancers [44]. The *NRG1* gene has been proposed both as a candidate oncogene and a candidate tumor suppressor gene [44]. Previous studies have shown that over expression of *NRG1* leads to the activation of *ERBB3/ERBB2* signaling and a poor prognosis. One of the reasons might be the paracrine effect of *NRG1* enhances the resistance to *RAF* and *MEK* inhibitors [45, 46]. However, no deletion alteration of *NRG1* has been reported in RMM previously. Further studies are necessary to explore the biological significance of *NRG1* deletion in RMM patients and how this affects the progression of melanoma. *BRAF* amplifications were also observed in this study. It is worth noting that *BRAF* amplification resulting in *BRAF* over-expression has been reported as one of the mechanisms responsible for acquired resistance to *BRAF* and/or *MEK* inhibitor [47, 48]. As has been documented, some common genetic variants found in MM, such as amplifications of *CDK4*, *MDM2* and *TERT* or deletions of *CDKN2A* and *ATM, * were rare in our RMM cohort [15, 30]. Therefore, these results may reveal a unique pattern of CNVs in RMM patients.

Rare gene fusions that have been reported in MM was also observed in our cohort. *NTRK* gene fusion has been reported in colonic MM [49] and could be a novel target of *NTRK* inhibitors [50]. Kim et al. identified a novel *ZNF767-BRAF* gene fusion that showed resistance to the BRAF inhibitor vemurafenib in respiratory MM patients [51]. Although these two fusion genes have been detected in our cohort, their biological mechanism remains unclear.

## Conclusions

To our best knowledge, this is the first study specifically for genetic alterations in Chinese RMM patients. We performed NGS analysis of 36 primary RMM specimens to describe the genetic characteristics and explore the pathogenesis of this rare tumor. Here, we confirmed that the genetic variation of RMM is different from that of CM, and reported the possibility of *BRAF* and *NRG1* as potential targets of RMM.

## Supplementary Information

**Additional file 1: Table S1.** Somatic mutations in 36 RMM.

**Additional file 2: Table S2.** CNVs and SVs variants in 36 RMM.

**Additional file 3: Table S3.** Univariate analysis to identify OS-related clinicopathological features.

**Additional file 4: Figure S1.** Distribution of RMM somatic mutations in the protein. Somatic mutation sites identified in our study were shown in functional domains, the amino acid length of Lollipop graph with indicates the number of mutations.

**Additional file 5: Figure S2.** The overview of identifying OS-related factors in 36 RMM patients. RMM patients were analyzed through the TSO500 pipeline and 388 mutations were identified. Univariate and multivariate regression analysis were performed to identify prognostic factors.

## Data Availability

The datasets used and analyzed during the current study are available from the corresponding author on reasonable request.

## Abbreviations

95%CI: 95% confidence interval
AJCC: American Joint Committee on Cancer
ARMM: Anorectal mucosal melanoma
CM: Cutaneous melanoma
CNVs: Copy number variants
EGF: Epidermal growth factor
FFPE: Formalin-fixed and paraffin-embedded
HR: Hazard ratios
IHC: Immunohistochemical
Mb: Megabase
MC: Melanocyte
MM: Mucosal melanoma
MSI: microsatellite instability
MSS: Microsatellite stable
NGS: Next generation sequencing
*NRGs*: Neuregulins
OS: Overall survival
RMM: Rectal mucosal melanoma
SNVs: Single-nucleotide variants
TMB: Tumor mutation burden
TSO500: TruSight™ Oncology 500
