# Supplementary material for: Genetic alteration of Chinese patients with rectal mucosal melanoma
Source: BMC Cancer. 2021 May 27;21:623. doi: 10.1186/s12885-021-08383-6 (PMC8161925; doi:10.1186/s12885-021-08383-6)
Supplement: Supplementary file 4 — Additional file 4: Figure S1. Distribution of RMM somatic mutations in the protein. Somatic mutation sites identified in our study were shown in functional domains, the amino acid length of Lollipop graph with indicates the number of mutations. [file 12885_2021_8383_MOESM4_ESM.docx]

***
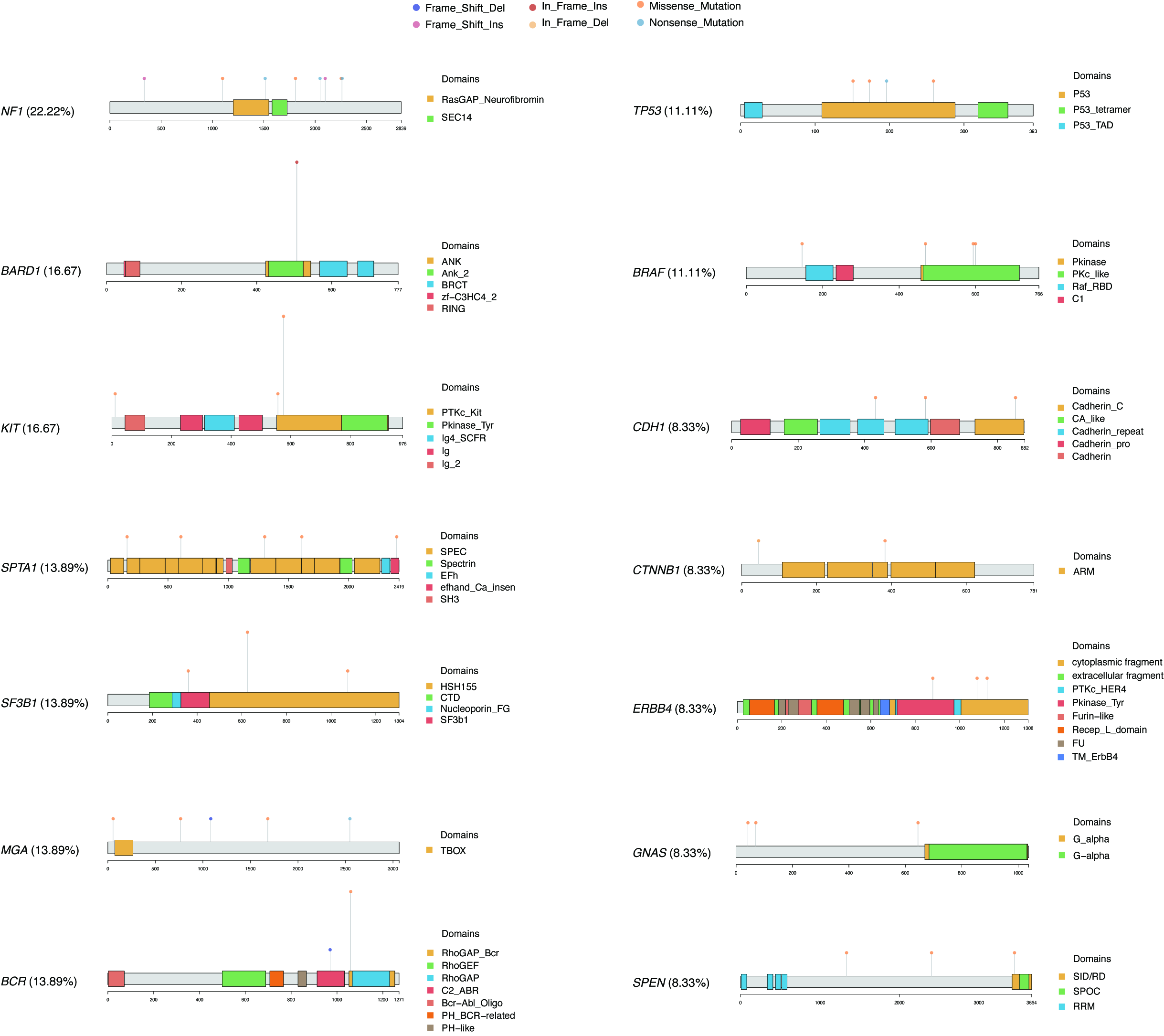
***

# Figure S1. Distribution of RMM somatic mutations in the protein. Somatic mutation sites identified in our study were shown in functional domains, the amino acid length of Lollipop graph with indicates the number of mutations.
