# Supplementary material for: Genetic alteration of Chinese patients with rectal mucosal melanoma
Source: BMC Cancer. 2021 May 27;21:623. doi: 10.1186/s12885-021-08383-6 (PMC8161925; doi:10.1186/s12885-021-08383-6)
Supplement: Supplementary file 5 — Additional file 5: Figure S2. The overview of identifying OS-related factors in 36 RMM patients. RMM patients were analyzed through the TSO500 pipeline and 388 mutations were identified. Univariate and multivariate regression analysis were performed to identify prognostic factors. [file 12885_2021_8383_MOESM5_ESM.docx]

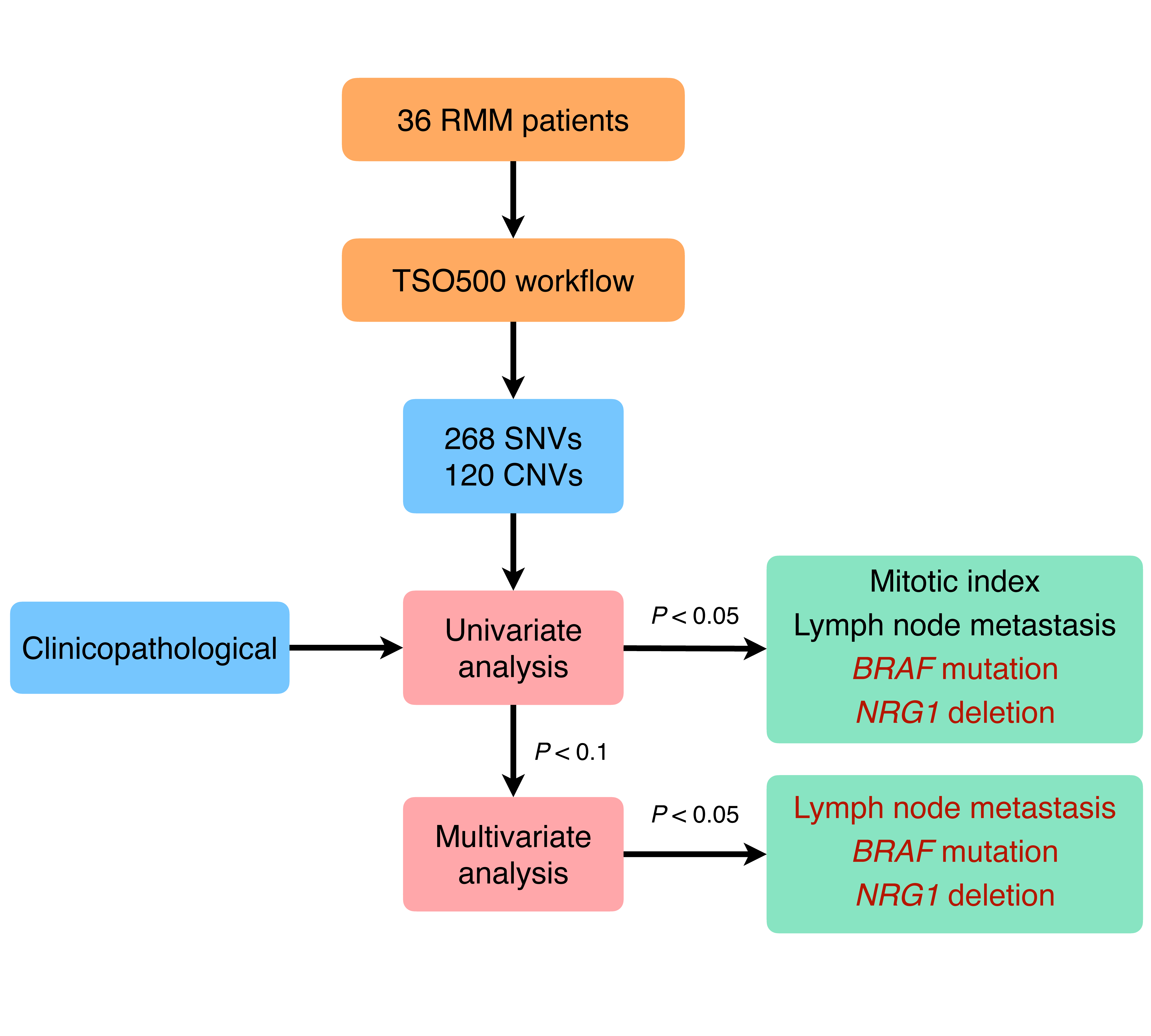
 **Figure S2.** The overview of identifying OS-related factors in 36 RMM patients. RMM patients were analyzed through the TSO500 pipeline and 388 mutations were identified. Univariate and multivariate regression analysis were performed to identify prognostic factors.
